# Making More of IT: Enabling Intensive Motor Cognitive Rehabilitation Exercises in Geriatrics Using Information Technology Solutions

**DOI:** 10.1155/2018/4856146

**Published:** 2018-11-01

**Authors:** M. A. McCaskey, A. Schättin, A. L. Martin-Niedecken, E. D. de Bruin

**Affiliations:** ^1^Research Department, Reha Rheinfelden, Rheinfelden, Switzerland; ^2^Department of Health Sciences and Technology, The Institute of Human Movement Sciences and Sport, ETH Zurich, Zurich, Switzerland; ^3^Department of Design, Subject Area Game Design, Zurich University of the Arts, Zurich, Switzerland; ^4^Department of Neurobiology, Care Sciences and Society, Division of Physiotherapy, Karolinska Institutet, Huddinge, Sweden

## Abstract

Although the health benefits of physical activity and exercise for older people are well established, a largely sedentary lifestyle still prevails in ageing western societies. Finding new ways to make exercise more accessible and acceptable for older adults must be developed to fully unleash its potential in preventing and weakening age-related physical and cognitive decline. Existing barriers to implement effective exercise-based treatment plans include motivational reservations on both the clinician's and patient's side, but also physical limitations caused by disease or deconditioning. Particularly in the more senior population, debilitating conditions do not allow adherence to currently recommended exercise regimes. A major rethinking of age- and user-adapted exercise is overdue. The high intensities required for physical and mental adaptations must be modifiable and personalized according to the functional status of each patient. Emerging information and communication technologies (ICT) have brought forward a plethora of attractive solutions for smart and adapted exercise, but there remains a vast gap between technological advancement and clinical relevance. Where in the beginning ICT for active ageing mainly focussed on aspects of usability and user experience, the current status of IT as applied in ageing populations noticeably shifted toward new services, applications, and devices that can be offered with the aim to prevent, compensate, care, and/or enhance daily life functioning of senior citizens. In this perspective paper, we aim to summarize the current state of the art in ICT-based interventions aimed at improved motor-cognitive control and make suggestions about how these could be combined with high-intensive interval exercise regimes to make rehabilitation for the impaired older adults more effective, and more fun.

## 1. Introduction

Driven by the observable patterns of ageing in western societies, attention towards healthy ageing as a means to preventing decline in functioning has been spreading rapidly over the last decade [1]. Today, the primary goal of public health is to increase the number of years of good health, and, thereby, to maintain independence and quality of life as long as possible. Healthy ageing is characterised by the avoidance of disease and disability, the maintenance of high physical and cognitive function, and sustained engagement in social and productive activities. These three components together define successful ageing [2]. In Europe it was the European Commission that identified active and healthy ageing as a societal challenge common to all European countries. Active ageing and independent living (http://ec.europa.eu/research/innovation-union/index_en.cfm?section=active-healthy-ageing) is one of the pillars around which actions are focused [3]. The specific action proposed to address issues related to this pillar is to develop Information and Communication Technology (ICT) solutions to help older people stay more active, and independently mobile for longer [4–6] and, by achieving that, contribute to Compression of Morbidity [7]. The importance of prevention of age- and behaviour-associated physical function decline and disabilities should therefore be in the focus of society's attention [8, 9]. Older adults are less likely to remain living independently in the community when they lose their ability to move and interact within their environments without needing assistance. Exhibiting higher rates of morbidity, mortality, health care utilization, and cost next to experiencing a poorer quality of life are other consequences [10, 11].

Furthermore, frail older adults and geriatric patients are particularly susceptible to loss of health-related (e.g., muscle size and, to an even greater extent, muscle power) [12] and skill-related fitness components (e.g., balance and reaction time) [13]. Notwithstanding the fact that physical exercise is considered one of the most effective nonpharmacological interventions to improve mobility and independence in frail older adults, no clear guidance regarding the most effective program variables exists [14]. Moreover, it is well established that geriatric patients are particularly sensitive to prolonged bed-rest; there are no clear exercise-based recommendations for multimorbid patients recovering from an acute illness. Even when patients are admitted to rehabilitation, the activity levels and exercise intensities administered are lower than would be required for actual improvement [15]. The quality of life and ability to function in daily activities of living rely on minimal levels of neuromuscular function and rapid loss of muscle power in hospitalised geriatric patients is often the beginning of years lived in frailty [16]. Sudden immobility due to nonavoidable hospitalisation has been shown to lead to a significant reduction of functional capacity and is associated with a substantial and sustained decline in functional status [17, 18]. Less than a fifth of hospitalised older patients with remaining impairments regain normal personal ability to perform the activities of daily living one year after discharge. About a third of discharged patients further deteriorate within a year and show debilitating signs of reduced cognitive and physical performance [16]. This has wide socioeconomic relevance, as the demographic changes come along with souring numbers of emergency admissions of patients older than 75 [19]. Almost half of geriatric patients admitted to emergency treatments, e.g., after falls or cardiopulmonary symptoms, have been reported to be rehospitalised within 6 months [19, 20]. Thus, finding ways to effectively prevent deconditioning leading to frailty and to significantly reduce the number of readmissions of older patients after a critical illness and subsequent discharge is of high relevance.

We are faced with the challenge of how to support public health policies that would help senior citizens in achieving the goals of primary and secondary (i.e., reducing readmission rates) prevention with the aim to remain independent. An extended life should ideally also involve preservation of the capacity to live independently and to function well [21]. For this purpose it is important that we understand the best ways of providing effective answers to the need for specifically tailored physical activities, for the provision of intellectual stimuli that keep older adults at highest possible levels of cognitive functioning, or for definition of supporting systems that facilitate social integration by keeping in close contact with family and peers.

Physical activity and exercise for older adults are highly recommended in order to promote maintenance of physical and cognitive functioning [22–26] at the highest possible levels. However, maintaining motivation to continue and adhere to exercise is often difficult as participating in conventional exercise may not have such an appeal [27]. There is, therefore, an identified need to perform more research aimed at establishing the best ways to encourage older adults to be more physically active in the long term [28]. Older adults may misinterpret exercising as being dangerous since perceived barriers are thinking participation increases the risk of having a heart attack, stroke, or death, despite the minimal likelihood of these occurring [29]. Barriers to physical activity resolve around major themes such as social influences (valuing interaction with peers, social awkwardness, encouragement from others, and dependence on professional instruction); physical limitations (pain or discomfort, concerns about falling, and comorbidities); competing priorities; access difficulties (environmental barriers and affordability); personal benefits of physical activity (strength, balance and flexibility, self-confidence, independence, improved health, and mental well-being); and motivation and beliefs [30]. This makes motivation and encouragement for exercise and physical activity fostered by professional support important. Finally, while we know that early aerobic exercise training after acute illness could enhance cardiovascular fitness and improve functional recovery [31], patients with functional impairment often cannot adhere to the commonly prescribed high-volume low-intensity exercises [32]. Providing short and effective exercise and increasing self-efficacy could prevent fear-avoidance strategies and help restoring prehospitalisation functional status.

Incorporating progressively intense but short exercise as part of a tailored and combinatory program has shown to be beneficial for healthy older men [33] and also feasible for comorbid geriatric patients [34]. Evidence from animal models and experimental research suggests a causal relationship of exercise intensity with physical [35, 36] and mental [37–39] health. Several reviews have summarized the evidence of the benefits of such high-intensity interval training (HIIT) for mental and physical health [37, 40, 41]. While these articles provide comprehensive recommendations for HIIT implementations and its limitations, they do not address the issue of how frail older adults can overcome physical impairments to benefit from this promising intervention. The main objectives of this perspective review are to (1) critically appraise the literature on aerobic exercise for healthy ageing and (2) highlight its limitations in practical translation of current recommendations. We then (3) review the evidence to illustrate how HIIT can overcome these limitations and what effects it may have on improved cardiovascular fitness and cognitive functioning. Next, (4) we illustrate how the effect of physical exercise on cognitive function and physical health can be boosted by combined motor-cognitive exercises. Finally, (5) we propose novel training solutions based on information and communication technologies (ICT) that exploit the versatility of enjoyable games to improve adherence and increase cognitive load during exercise (i.e., exergames). The working hypothesis (6) is that combined intensive cognitive and physical exercise delivered as smart ICT-solutions has the potential to dampen the cognitive decline observed in older people in the early-to-middle stages of dementia [42] next to improving their physical functioning [43, 44]. We intend to illustrate how a successful implementation of these modern technologies in combination with short intense exercise bouts can help to make more of the little time invested in primary and secondary prevention.

## 2. Aerobic Exercise for Healthy Ageing: A Critical Appraisal of the Literature

Physical activity has been shown to be vitally important for maintaining endurance, strength, and function in older adults [45]. Optimisation of physiological function throughout the lifespan has been advocated as an important part of any contemporary biomedical policy addressing global ageing [46]. Reductions in both muscle volume and oxidative capacity per volume in the course of ageing result in declined oxidative capacity and appear to be an important determinant of the age-related reduction in aerobic capacity [47, 48]. These reductions are believed to explain the reduction in preferred walking speed in healthy older subjects, which requires a higher fraction of ventilatory threshold [49], which is also associated with cognitive [50] and mobility [51] decline.

Decay of cognitive performance in old age is predominant in most individuals, an observation confirmed, for example, by a large Italian epidemiological study demonstrating that ageing associated cognitive decline has a prevalence rate of 28% for people from 65 to 84 years of age, and cognitive decline without cognitive complaints summed up to 45% of people showing some kind of cognitive impairment without dementia [52]. Worldwide, the number of people living with dementia is estimated to be 47.5 million and by 2050 this figure will triple to 135.5 million [53]. Dementia is a broad category of brain diseases causing a long-term and often gradual decrease in the ability to think and remember and that is great enough to affect a person's daily functioning [54]. Although the signs and symptoms linked to dementia can be understood in three stages, training interventions ideally should address older people in the early-to-middle stages of dementia [55].

Research has pointed out recently that physical activity may be relevant for healthy brain ageing and may protect from cognitive decline and dementia [56–60]. Most physical intervention studies, which focused on adaptations in cognitive performance, brain function, or brain structure, applied aerobic-type exercise. Two meta-analytic studies reported that aerobic exercise is effective in increasing cognitive performance in general and executive function in particular [61, 62]. Studies that are more recent also found that strength and coordination training might positively affect cognitive abilities [63, 64]. Voelcker-Rehage et al. [65] demonstrated training specific functional plasticity in the brain based on functional magnetic resonance imaging data. It appears that aerobic training increases activation in the sensorimotor network and coordination training leads to a higher activation of the visuospatial network, whereas strength training changes the hemodynamic activity of brain regions associated with response inhibition processes.

Current recommendations for exercise prescription to reverse frailty follow the Frequency-Intensity-Time-Type training principles [66]. According to the literature, a frequency of 2-3 exercise bouts per week at progressive moderate to vigorous intensity is recommended [67]. The intensity has been further specified as 70% to 75% of the age-adjusted maximal heart rate [68]. As the review by Bray et al. [66] points out, duration should be adapted according to the frailty status, age, and consistency of exercise participation. Ideally, frail adults should train for 30-45min while prefrail adults should train for 45-60min. For frail older adults, a shorter duration is recommended as they may get easily fatigued [69]. The exercise should not be limited to one type of intervention. Rather, a multicomponent regime with different exercise components has shown to be most accepted and effective in older adults [66, 70]. Any program should, however, include and start with an aerobic warm-up component [66], which can also just be brisk walking [71]. The greatest unknown in exercise prescription for the older adults is the progression rate of intensity. To really benefit from any kind of exercise, tailored progression to reach and extend the individual's capacity limits is of particular importance. Only by continuously monitoring each patient's capacity and adapting the training intensity can long-term benefits be expected [66]. Meanwhile, for healthy older adults, the American College of Sports Medicine and the American Heart Association (2007) recommend moderate-intensity aerobic physical activity five days a week or vigorous-intensity on three days per week. Additional strength and flexibility exercises are recommended at least twice a week with incorporation of balance and coordination exercises to reduce risk of injury form falls [72].

## 3. Limitations in Practical Translation of Current Recommendations

The aforementioned recommendations for older adults demand physical activity on most or all days of the week. However, only 31% of persons, 65 to 74 years, report regularly engaging in moderate physical activity for 20 minutes or more three days a week [73]. By 75 years of age, the rate drops to a low level of 20% [73]. Furthermore, only less than 5% of older adults (60+ years) are physically active in terms of meeting the recommended 30 min a day [74, 75].

A reason might be that older adults may not be able to initiate physical activity at recommended levels [76]. A possible solution could be that, through appropriate and progressive increase in training amount, the recommendation for older adults can be achieved in rehabilitation or exercise settings [76]. Nevertheless, for many older adults with physical, cognitive, or combined physical-cognitive impairments the execution of physical exercise according to the recommended levels may never be possible [76]. Therefore, a need exists for innovative ideas and to develop training interventions that support physical and cognitive functioning and that facilitate exercise participation by considering holistic approaches (physical, cognitive, and social), current physical and mental status (user's profile), next to shorter training time requirement, and the consideration of innovative new technologies-based solutions [22]. At the moment, two interesting training approaches are discussed in the research field of physical activity in older adults: high-intensity interval training [77] and motor-cognitive training [44].

## 4. Higher Intensities Intervals for Better Efficacy?

The rise in research activities on the topic of high-intensity interval training (HIIT) is striking. The last decade has seen a more than tenfold increase in publications looking into the effects and mechanisms of HIIT. It has since been shown that HIIT is more effective at improving cardiovascular and cardiopulmonal function when compared to moderate intensity continuous training regimes [78]. Several individual studies with varying training modes and reviews with meta-analyses have confirmed these findings and also uncovered a row of unexpected beneficial effects on bodily functions: if properly implemented (e.g., 4 × 4 minutes of HIIT over at least 12 weeks), it has the potential to change molecular blood composition, reducing cardiovascular disease risks (e.g., reduction in blood pressure), and improving insulin sensitivity [32], and increase brain derived neurotrophic factors [37]. Importantly, HIIT has also undergone safety evaluations in animal and human studies, consistently reporting its safety, even in patients with cardiac or metabolic dysfunctions [79]. A study by Rosendahl et al. [80] demonstrated positive long-term effects in physical function of a high-intensity exercise program for older persons dependent in activities of daily living, of whom most had severe cognitive or physical impairments. No adverse events occurred in the study, despite the high prevalence of different diseases. HIIT training is also amenable with novel technologies [81]. A point that needs attention, however, is determination of the optimal dose-response for interval training. The studies discussed so far used different protocols with different intensities which may result in differing responses of the trainees [82]. Modulating the parameters of intense exercise using additional cognitive load and smart ICT solutions may provide a more potent stimulus for synaptic adaptions and potentially translate into improved physical function [83]. Moreover, if ICT based solutions can integrate the individual's vital signs and relevant biomarkers to adapt the training, safety and efficacy may also be improved. If, for instance, new technologies would allow surrogate measurement of oxidative-nitrosative-inflammatory stress during or before exercise, the exercise intensity could be adapted to prevent harmful blood-brain-barrier disruptions and potential maladaptation [37].

HIIT involves repeated bouts of high intensity effort followed by varied recovery times [84]. According to the American College of Sports Medicine (ACSM), each bout ranges from 5 seconds to 8 minutes performed at 80% to 95% of a person's estimated maximal heart rate. The ACSM recommends recovery periods to be performed at 40% to 50% of a person's estimated maximal heart rate [84]. This intermittent recovery phase allows untrained people with lower functional capacity, e.g., older adults [85], to work out harder than during continuous low-intensity exercises [86]. It has been labelled with “higher efficacy, tolerability, and adherence without compromising patients safety” [36]. Adapted to the capacity of the patients, HIIT presents a viable alternative to poorly adhered low-intensity exercise to reduce the risk for inactivity-related disorders [87]. However, recent concerns regarding the underinvestigated risks on brain health have given rise to more critical voices [37, 41]. One review found a slightly higher incidence of adverse response during or shortly after HIIT exercise in patients with cardiometabolic diseases [41]. Therefore, despite all the benefits of HIIT, the precautions taken before employing a HIIT program for patients should be taken at least as strict as before any other form of exercise. Patients must be stable and medical monitoring should be provided during the exercise.  Table 1 shows an overview of selected high-intensity interventions for older people. Selection criteria were studies investigating HIIT in participants older than 65 years.

Adapted physical activity at high intensity is capable of counteracting age-related changes and performance loss not only in the cardiovascular and muscular system [91], but also on cognitive performance and brain structures [92]. Intense physical activity clearly improves cerebral blood supply and increases neuronal activity, which has been shown to stimulate neurogenesis [37]. The predominant hypothesis regarding this effect is that brain-derived neurotrophic factor (BDNF) is upregulated with higher protein concentrations observed in brain regions particularly important for learning and memory (e.g., hippocampus). BDNF plays an important role in neural plasticity and has been associated with cognitive performance in healthy and clinical populations [39, 93]. Just 20 minutes of HIIT has shown to increase BDNF serum levels with a relevant functional effect, i.e., to influence behavior and cognition [36].

A review of the literature by Penrose et al. suggests that, in order to better harvest the effects of exercise on cognitive function in patients with dementia, physical activity should be combined with cognitively stimulating activities [80, 94]. The next section takes a closer look at how current exercise regimes are employed in combination with cognitive loadings to improve mental and physical capacity.

## 5. Motor-Cognitive Exercise

Cognitive training studies have often shown highly task specific effects [95–99]. More widespread transfer effects were found when different cognitive abilities were combined in complex interventions or lifestyle changes [100]. Nevertheless, effects were often small, while aerobic training elicited both broad transfer and relatively large effects. These findings led to the assumption that not only the combination of different cognitive abilities, but also the combination of cognitive and physical training improves cognitive performance in old age to a greater extent than the training of an isolated ability [42, 101–104].

Most daily activities are an interaction of motor and cognitive functions. From an evolutionary point of view, tasks were always an interconnection of motor and cognitive functions [105]. However, training interventions of older adults are usually split into a physical and a cognitive part. Studies demonstrated that larger benefits could be induced by the combination of motor and cognitive exercise as the combination might generate more synergistic beneficial changes [101, 106]. Based on reports found in the scientific literature, the cognitive supplements should be selected such that they share similar mechanisms with exercise and, thus, theoretically have the potential to support the action of the physical exercise part [42] or vice versa. Moreover, an indication of previous studies was that the combination has to be simultaneously to evoke positive effects [107]. Therefore, a need exists to take both components into account to design effective interventions and to design multimodal interventions [101], c.f. with the help of exergames [83].

Environmental challenges cause plastic adaptations of the human brain. Physical and cognitive exercise might trigger similar neurobiological mechanisms that go hand in hand to induce plastic changes in the brain [106]. Physical activity seems to facilitate neuronal plasticity by upregulation of blood circulation, neurotrophic factors, neurogenesis, and synaptogenesis [107–109]. The concurrent cognitive stimuli might activate the synapses and this in turn supports the survival of newly generated synapses and their integration into preexisting neuronal networks [106, 110]. Moreover, motor-cognitive exercise might also improve the synapse communication in brain networks that are responsible for movement coordination and execution because of the motor stimuli. This in turn could positively influence the communication from the motor area of the brain to the muscles. An efficient brain-body communication might contribute to motor performance (e.g., balance and strength). Furthermore, adaptions in the muscles and in the cardiovascular system might depend on the movements that are executed for the motor part.

Various applications exist that include the training of motor and cognitive abilities. Dancing and Tai Chi are examples of motor-cognitive exercises. Dance movements and Tai Chi both combine physical activity, sensorimotor interaction, and cognitive functioning in a possible social environment. Studies showed that dancing has positive effects on different cognitive functions, e.g., reaction time, working memory, and flexible attention, and on lower body muscle endurance, strength, and balance in older adults [111–114], however, only when an appropriate dose level of dancing training is reached [115]. Also Tai Chi performance showed positive effects on physical functions and ameliorated cognition in older adults [116–118].

More recently an emerging complementary tool in exercise and rehabilitation in the older population has been exergames [119]. Exergaming combines motor and cognitive exercises as video games that have to be played by being physically active. The exergames used in research range from commercially available games (Nintendo Wii Fit, Xbox Kinect, and Dance Dance Revolution) to purpose-developed systems. Exergame studies demonstrated positive benefits for cognition and/or physical functioning in older adults [42, 120–124]; however, the training principle of specificity seems to apply to exergaming. The choice of exergame console [125] and game content used for training [126] influences the areas of (postural) control trained and improved. Which motor and cognitive functions will be or can be improved and triggered through exergaming most likely also depends on the consideration and implementation of training principles (e.g., intensity, frequency, and user-centred).

## 6. Personalized ICT Interventions for Active Ageing

In this section we will explore how the societal challenge of active and healthy ageing that is common to all European countries might be approached through the purposeful development of smart ICT technologies. We will begin by discussing general solutions that address behaviour associated and physical function decline with age [8, 9]. We will then put our focus on existing solutions that incorporate HIIT training [127].

In parallel to the push by scientists, industry leaders, and government officials to make medicine more personalized [128], there is a call for development of preventive physical activity strategies towards personalized exercise programs. In this context, ICT solutions have been reported as promising [129, 130] and exergames lend themselves to personalization of interventions [131]. Various ICT based solutions for optimised tailoring of exercise interventions have been proposed to support not only people with dementia but also their families and caregivers [132, 133]. Video game play-driven physical activities require mental engagement and would, thus, be in line with recommendations of the Global Council on Brain Health [134].

Various ICT-based applications for geriatric physical and mental training or therapy with older adults as well as specific recommendations for the design of these solutions are available; see [133] for a comprehensive overview. ICT may serve, for example, to mitigate some of the negative side effects of ageing (e.g., physical and cognitive decline) but can also serve to develop new capability enhancing opportunities. Where in the beginning information technology for active ageing mainly focussed on aspects of usability and user experience, the current status of IT as applied in ageing populations noticeably shifted toward new services, applications, and devices that can be offered with the aim to prevent, compensate, care, and/or enhance daily life functioning of senior citizens [133].

**Figure 1:**
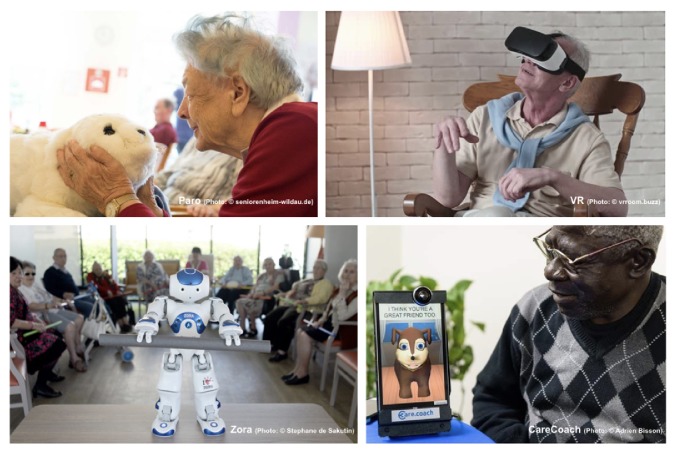
Selection of ICT-based solutions for seniors.

The Human-Computer-Interaction and the game design communities provide useful research and development examples that integrate effective exercises and training for older adults in an attractive and motivating multimedia design. The spectrum includes a wide range of innovative applications supporting patients and their relatives as well as caregivers (Figure 1). Well known examples include the social robots, like Zora or Paro. While social robots are designed to facilitate social interactions between patients as part of a multisensory behaviour [135], virtual coaches (e.g., CareCoach) use motivational cues for older people to maintain daily activities [136]. While such solutions provide some form of companionship and monitoring, they do not engage users in any meaningful physical activities. So-called social exergaming solutions, on the other hand, include joyful and purposeful physical activity while simultaneously stimulating social interaction to counter isolationism and loneliness [137].

### 6.1. Exergames for Older People

**Figure 2:**
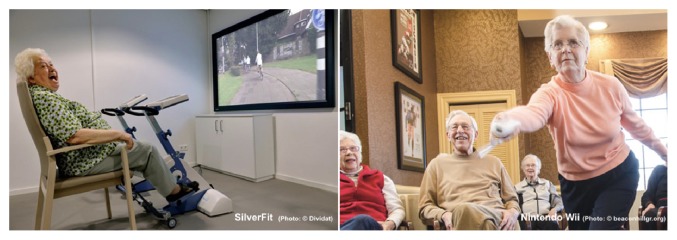
Selection of specifically developed (left) and general game market (right) commercially available exergames for seniors.

Exergames (Figure 2) are games, which are controlled by physical activity and body movements of the player (exertion/exercise + gaming) [138]. Exergames have been approved to be a suitable tool, which, if designed properly, engages seniors in physical activity in an effective (by integrating different training aspects and principles) and attractive manner (by implementing motivational designs). Depending on the virtual game scenario, the required in-game tasks, and the respective movement-based controller system (e.g., haptic or gesture-based input devices), different motor-cognitive functions and other health parameters can be improved when playing exergames.

### 6.2. High Intensity Interval Exergames for Older People

**Figure 3:**
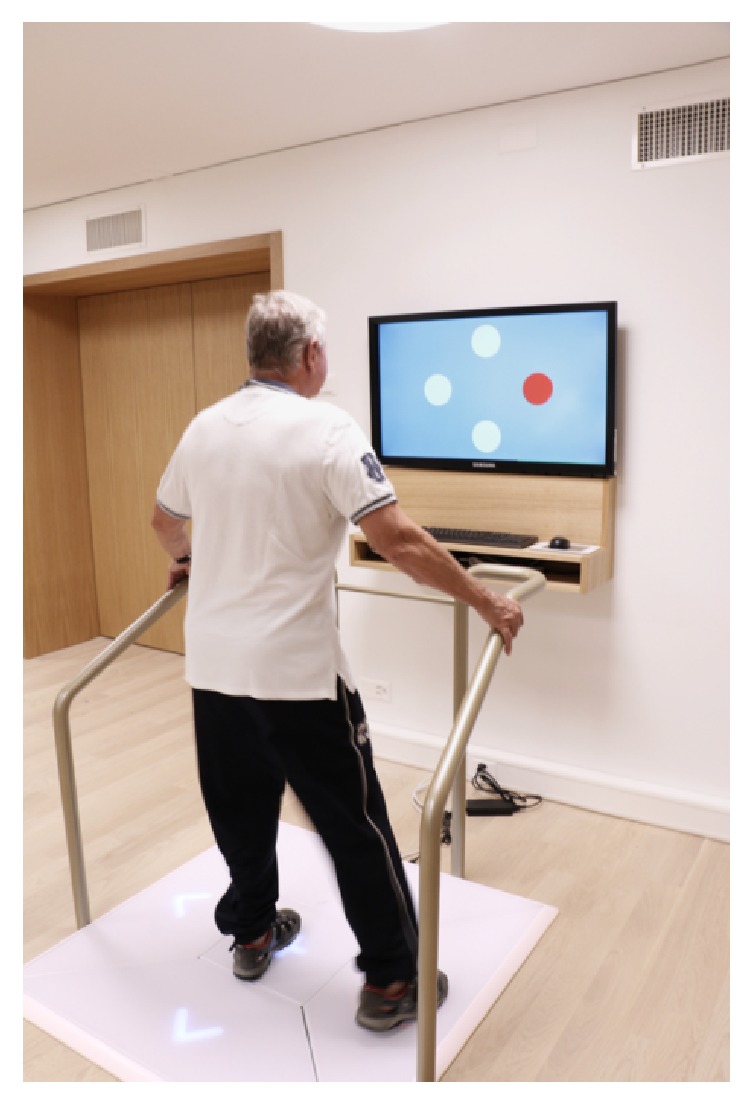
Dividat Senso plate with HIIT module for seniors [127].

Gerontology research has provided insights into the impact of exergames-based interventions on seniors' motor-cognitive abilities [42, 120, 121, 123, 124]. A recently published, comprehensive systematic review provides an extensive overview of existing studies with exergames and their effects on older adults' level of physical activity [139]. Their findings support the usage of exergames in older adults, as shown by increased motivation and engagement of older people in general physical activity. Further systematic reviews have summarised evidence supporting the effectiveness of exergames to treat age-related chronic disease in terms of physical, psychological, and cognitive rehabilitative outcomes [140, 141]. However, research of exergame effects on aerobic capacity is largely missing. Currently, it is assumed that exergames are merely able to elicit light intensity energy expenditure when employed in community-dwelling older people [142]. But exergaming has also been described as an acceptable, safe [143], and innovative way to design enjoyable high-intensity interval training [81]. Only very few studies and game developers have specifically considered aerobic training in general [119] and HIIT specifically for seniors. To the best of our knowledge there are three studies examining HIIT and exergaming: one study used a boxing approach in young adults and adults [144], another study was performed with young adults on ergometers [81], and one program was designed for older adults that has to be performed in standing position on a pressure-sensitive plate [127] (c.f.  Figure 3). The latter study was performed at the Institute for Human Movement Sciences and Sport (ETH Zurich) while the other two studies were found by a literature search on Scopus using the keywords exergaming and high-intensity interval training.

### 6.3. Sport Scientific Recommendations for Personalized Exergames for HIIT and Motor-Cognitive Training in Older People

Based on theoretical considerations [145] combined with exercise recommendations for older adults [22] and knowledge from an effectively implemented HIIT training in older adults [146], an ergometer-based exergame HIIT program could be designed that considers 4x4 minutes >90% of peak oxygen consumption [VO_2  peak_] with 3 minutes' pedaling at no load on an ergometer on 3 days per week. The training can be extended with 2 days per week treadmill walking (45 minutes at 70% of VO_2  peak_). Before each activity, there is 5–10 min of warm-up and cool-down activities. Exercises for older adults should consider a Rate of Perceived Exertion 11–14 (6–20 scale). Furthermore, exercise with integrated cognitive challenges should target executive functions (e.g., inhibition, working memory, and flexibility) and White Matter Hyperintensity (WMH; Figure 3) [120, 121, 147]. Cognitive stimuli can be presented via visual (e.g., frontal screen) [120, 121] or acoustic (e.g., music player) systems [148] whereby the trained cognitive function determines the composition of the stimuli (e.g., inhibition: participants have to react to key stimuli and suppress reactions to other stimuli). Reactions can be recorded via the training device (e.g., step sequences, see Figure 3), pressing a button, or speech sounds depending on the wanted accuracy and objective. For a HIIT training, it might be reasonable to adjust the intensity of the cognitive load to the physical load of the exercise (e.g., more stimuli during the low-intensity exercises phases) to achieve an optimal challenge [149]. An optional complementation could be pedometers to increase lifestyle physical activity. Training program duration should be 6 weeks to see an effect in measures of aerobic capacity [146] and 12 weeks for changes in WMH with task-oriented training in standing position and targeting timing and coordination of gait [150].

Considering the above-mentioned points, a program that consisted of an exergame-based HIIT performed while standing on a pressure sensible platform (Dividat Senso Step Plate, Schindellegi, Switzerland, 93/42/EWG certified) and cognitively interacting with an on-screen scenario with audio-visual feedback might be designed as follows: short intervals of higher intensity exertion (fast alternating steps to keep a rocket in the air; 70-90% of maximum heart rate (HRmax)) alternated with active rest periods (50-70% of HRmax) for a total of up to 25 minutes. Important in this context is the reliable determination of HRmax values. Where the rating of perceived exertion using the 20-point Borg-scale seems valid and reliable for older adults [151], it seems important not to underestimate the true level of physical stress in older trainees in relation to reaching the appropriate intensity of exercise [152]. The intervention should be performed individually three times per week (to be in line with exercise recommendations related to frequency of training) for a period of four weeks with twelve sessions. Each training session is partitioned into three parts: five minutes' warming-up on a cycle ergometer at 50-70% of HRmax, followed by up to 25 minutes of HIIT, using different exergames on the pressure sensible platform. The sessions are finished with a cool-down of five minutes on an ergometer. Participants are expected to complete twelve training sessions while being monitored by a trainer, who systematically observes and supervises participants throughout the training. The program is individually tailored to each participant according to a training protocol based on the guidelines published by the American College of Sports Medicine [153] and is aimed at increasing endurance capacity.  Table 2 includes an exemplified HIIT protocol that can be included into the exergame training program.

## 7. A Call for More HIIT in Exergames for Older Adults

Our review revealed that there are currently only two exergame HIIT training programs [81, 127] and only one that is specifically designed for older adults. Following and based on our longstanding experience in designing and researching holistic, research-based and user-centred state-of-the-art exergames for and with various, heterogeneous target groups, we aim to contribute to the ongoing debate and make some recommendations for the design of personalized exergames for HIIT and rehabilitation in seniors.

### 7.1. Interdisciplinary Collaboration

To create a holistic and user-centred design, it is recommendable to work with interdisciplinary developer teams of experts from all related fields. In the context of personalized exergames for HIIT and rehabilitation in seniors, the team should consist of older people, therapists, movement and sports scientists, game designers, and industrial and interaction designers as well as Human Computer Interaction researchers, who can provide relevant knowledge from different perspectives.

In this scenario, the targeting group (older people, therapists, and relatives) is part of the developer team and is directly involved in the participatory and user-centred design process. It can be further distinguished between the first stage- (seniors, who will play the game as single and/or multiplayer game) and the second-stage-targeting group (therapists and relatives, who will attend the senior while playing and/or could be involved in the gameplay).

### 7.2. Design Recommendations for Exergames in Older People

#### 7.2.1. Multilayered Design

**Figure 4:**
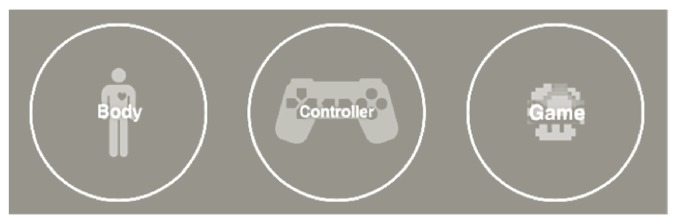
Three levels of research-based and user-centred design in exergames (source: Martin-Niedecken, 2017).

The holistic design process should focus on three design levels, which need to be covered symbiotically and in relation to one another (Figure 4).

1. Body: targeting group-specific, physical requirements need to be taken into account and specific training/exercise concepts (adapting to the training progress) must be developed including the individual and social body as play element. In the context of HIIT and motor-cognitive training, designers should especially focus on movements from traditional HIIT and motor-cognitive training/rehabilitation interventions and take or modify these into game-related input movements.
2. Controller: choose and experiment with targeting group-specific game devices such as specifically developed input devices and/or therapy/training devices, which are used to transfer the player's body movements to the game and which could provide an additional playful experience beside the virtual game scenario. Equally to the body design level, it is important to think about devices, which allow for the implementation of different motor-cognitive stimuli as well as for HIIT movements and which easily can be integrated into the specific body movements rather than disturbing the flow of movements and gameplay.
3. Game: the game should include varied and balanced game mechanics, feedback, aesthetics, dynamics, story, and sound, which suit the targeting group's requirements and preferences. Furthermore, it is important that the game takes up the expert knowhow on specific useful cognitive stimuli and HIIT and provide a playful representation and a well-balanced feedback loop for the player.

Furthermore, in the context of geriatric rehabilitation, it is very useful to feature an additional tool for the therapists and/or caregivers, which allows organizing and evaluating training sessions and plans. Such a therapist-UI could also be used to manually adapt specific in-game parameters as well as controller settings and input movements to the current physical and emotional needs and states of the patient during an exergame-based training/therapy session.

To sustainably enhance the effects of exergame training/therapy and support adherence of seniors in playful exercising, designers should create applications which can be used in a clinical setting and in the presence of a therapist or caregiver, as well as applications for the home use, which are easily accessible and can be used alone by older people.

#### 7.2.2. Multilevel Process: Design, Evaluation, and Redesign

Following a traditional user/player-centred game design approach [154], developer teams should always start with a review (and testing) of related R&D work on all three design levels and use the gained knowledge as base for their first concepts.

Among others, the designs of all elements related to the three design levels should be based on theories and approaches like the “game flow” [155] and the “dual flow” [156] to allow for a maximum attractive and effective training/gameplay experience through an individually adaptable game difficulty and complexity and in terms of a multiplayer, a dynamic grading, and balancing of all players even if they have different skills [157]. Additionally, findings from the interdisciplinary research debate with and on exergames provide further insights into the impact of various controller technologies [158], body movements [159], social exertion, and bodily interplay [160] as well as various design parameters (visuals, audio, perspectives and points of view, etc.) on the player's gameplay/training experiences. This knowhow should also be taken into account, when designing a targeting-group specific exergame.

Another promising and valuable approach is the involvement of older adults' relatives and especially of their grandchildren into a multiplayer game setting [161]. An intergenerational design approach can allow for new (social) interaction mechanisms and holds great potential for future exergame developments for seniors. Furthermore, experimenting with mixed realities (augmented and virtual realities) and/or mixed genres (exergames meet strategy games) in could open up new design patterns.

By involving the targeting group(s) into the design process from the very beginning, designers can easily gain valuable insights into personal preferences, needs, and requirements and enhance identification of their targeting groups with the final product.

To start working with the targeting group(s), powerful and effective methods are guideline-based focus group interviews and the use of first draft paper prototypes, sketches, movement concepts, and input device prototypes as inspiration and basis for the discussion. Developer teams should run several user-tests and collect the feedback from all user perspectives (e.g., older people, therapists, and relatives).

After the first explorative phase, developers should start implementing first design drafts and let their targeting group(s) test them again immediately to get feedback, which could then be implemented in the further development of various or individual concepts.

As soon as a holistic prototype is ready after some design iterations, developer teams should run first user studies on specific research questions to gain deepened insights and knowledge on the attractiveness and effectiveness of their exergame. Again, the results will be used as basis for the redesign. This dynamic process can be repeated several times until the final product is ready.

So far, and to the best of our knowledge, there are any exergame projects or products for HIIT or motor-cognitive training/rehabilitation in seniors, which focused on this exact holistic and symbiotic approach.

**Figure 5:**
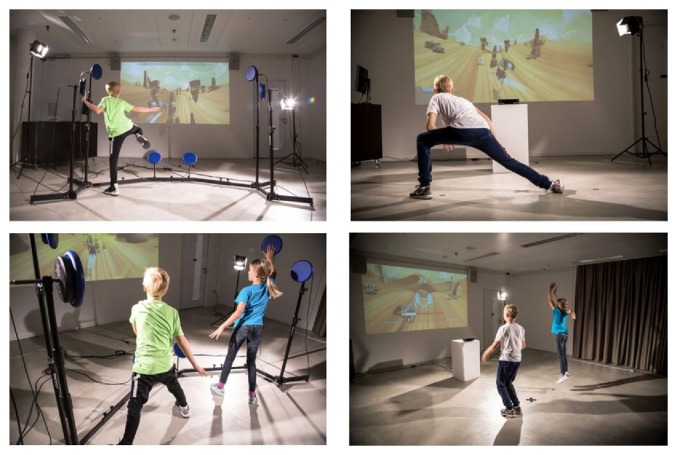
User-centred and adaptive single and multiplayer exergame environment “Plunder Planet,” which was developed with and for children and young adolescents which can be played with different motion-based controller devices challenging the motor-cognitive, coordinative, and endurance abilities of the players [149].

However, in current studies, Martin-Niedecken and colleagues could show the positive impact of an adaptive and user-centred exergame training environment on dual flow, motivation, and training performance of children with and without previous gaming and sports experience [162–165]. The fitness game “Plunder Planet” (Figure 5) was developed following an iterative, research-based, and user-centred design approach like outlined above.

## 8. Conclusion

To conclude, we would like to point out the need for future projects and works, which take these guidelines and recommendations into account and, through the implementation of personalized, state-of-the-art designs and the interdisciplinary collaboration, help to improve the feasibility and acceptance of exergames as a beneficial tool in the context of geriatric training and rehabilitation in general as well as for exergames for HIIT training.

Public policy makers bear responsibility in the context of supporting healthy ageing [166] and must recognise the needs for prevention by working towards environments, societies, and building infrastructure that facilitate active living of seniors. Innovative public health policy should allocate attention to technology, in terms of advanced instruments for healthcare and in terms of support that can be provided to supporting performance of everyday activities of older individuals. Although technology is permeating our everyday lives, most solutions target tech-savvy people, e.g., persons “well informed about or proficient in the use of modern technology, especially computers [https://en.oxforddictionaries.com/definition/us/tech-savvy],” and do not specifically focus on older users [166].

## Figures and Tables

**Table 1:** Overview of selected HIIT studies for older people (>65 years).

Reference	Intervention	Outcomes	Main finding
Rosendahl et al., 2006 [80]*∗*	C=inactive controls F=29 within 3 months I=”high intensity” T=n/a T= HIFE (functional exercise at high load) + Protein supplementation	Berg Balance Scale Max Gait Speed 1 rep max (lower extremities)	*⬀* *⬀* *⬀*

Rosendahl et al., 2008 [88]*∗*	C=inactive controls F=29 within 3 months I=”high intensity” T=n/a T= HIFE	Fall rate	=

Littbrand et al., 2009 [89]*∗*	C=inactive controls F=29 within 3 months I=”high intensity” T=n/a T= HIFE	Barthel ADL	*⬀*

Saucedo Marquez et al., 2015 [36]	C=continuous exercise at moderate intensity (70% HR_max_) F=one-time intervention I=90% HR_max_ and recovery intervals 20% HR_max_. T=20 minutes T= HIIT on cycle ergometer with intervals of 1 min duration.	BDNF serum levels	*⬀*

Wisloff et al., 2007 [90]	C=continuous walking at 70% HRpeak. F=3/week I=95% HR_peak_ and recovery intervals at 50% to 70% HR_peak_. T=20 minutes. T= 4 minutes HIIT with 3 minutes recovery interval.	Flow-mediated dilation Muscle biopsy (Mitochondrial function) Quality of life VO_2peak_	*⬀* *⬀* = *⬀*

An overview of a selection of studies investigating types of high-intensity interval exercise in older people: C=comparator; F=frequency; I=intensity; T=duration/time; T=type; HR=heart rate; BDNF = brain-derive neurotrophic factor; ADL = activities of daily living; *⬀*: in favour of intervention; *⬂*: in favour of comparator; =: no difference between groups.*∗*: based on the same interventions with different outcomes.

**Table 2:** Example of a theory-based HIIT training protocol schedule designed for the Dividat Senso plate (Figure 3).

Week 1 (Trainings 1-3)	High-intensity interval: 4x1 min up to 9x1 min at 70-80% of HRmax+10%, Borg 12-14	If participant was already working at ~80% level of HRmax+10% at the end of week 1, keep continuing at ~90% level of HRmax+10%
	Active recovery interval: up to 8x2 min at 50-70% of HRmax+10%	

Week 2 (Trainings 4-6)	High-intensity interval: 3x2 min up to 6x2 min at 70-80% of HRmax+10%, Borg 12-14	If participant was already working at ~80% level of HRmax+10% at the end of week 2, keep continuing at ~90% level of HRmax+10%
	Active recovery interval: up to 5x2 min at 50-70% of HRmax+10%	

Week 3 (Trainings 7-9)	High-intensity interval: 3x2 min up to 6x2 min at 80-90% of HRmax+10%, Borg 15-17	
	Active recovery interval: up to 5x2 min at 50-70% of HRmax+10%	

Week 4 (Trainings 10-12)	High-intensity interval: 5x2 min up to 8x2 min, at 80-90% of HRmax+10%, Borg 15-17	
	Active recovery interval: up to 7x1 min at 50-70% of HRmax+10%	

The participant performs alternating steps on the plate and, by doing that, keeps a rocket above a certain threshold line. The trainee may face different cognitive challenges by having to control the rocket along a straight line in the air using different stepping speeds or through having to manoeuvre the rocket in between obstacles.
